# Molecular mechanisms of exercise intervention in alleviating the symptoms of autism spectrum disorder: Targeting the structural alterations of synapse

**DOI:** 10.3389/fpsyt.2023.1096503

**Published:** 2023-03-31

**Authors:** Wenhao Zong, Xiaowen Lu, Guijun Dong, Li Zhang, Kefeng Li

**Affiliations:** ^1^School of Exercise and Health, Shanghai University of Sport, Shanghai, China; ^2^Department of Sports, Quzhou University, Quzhou, China; ^3^College of Sports and Health, Shandong Sport University, Jinan, China; ^4^Guangdong-HongKong-Macau Institute of CNS Regeneration, Jinan University, Guangzhou, China; ^5^Department of Medicine, Quzhou College of Technology, Quzhou, China

**Keywords:** autism spectrum disorder, ASD symptoms, exercise intervention, molecular mechanisms, synaptic structural plasticity

## Abstract

Autism spectrum disorder (ASD) is a complex and heterogeneous neurodevelopmental disorder characterized by stereotyped behaviors, specific interests, and impaired social and communication skills. Synapses are fundamental structures for transmitting information between neurons. It has been reported that synaptic deficits, such as the increased or decreased density of synapses, may contribute to the onset of ASD, which affects the synaptic function and neuronal circuits. Therefore, targeting the recovery of the synaptic normal structure and function may be a promising therapeutic strategy to alleviate ASD symptoms. Exercise intervention has been shown to regulate the structural plasticity of synapses and improve ASD symptoms, but the underlying molecular mechanisms require further exploration. In this review, we highlight the characteristics of synaptic structural alterations in the context of ASD and the beneficial effects of an exercise intervention on improving ASD symptoms. Finally, we explore the possible molecular mechanisms of improving ASD symptoms through exercise intervention from the perspective of regulating synaptic structural plasticity, which contributes to further optimizing the related strategies of exercise intervention promoting ASD rehabilitation in future.

## 1. Introduction

Autism spectrum disorder (ASD) is a group of common, complex, and highly heterogeneous neurodevelopmental disorders characterized by the core symptoms including deficits in social interaction and communication and repetitive and stereotyped behaviors, interests, or activities (1). The worldwide prevalence of ASD is approximately 1%, while this estimate is much higher in high-income countries, such as the United States (2). Together with these core symptoms, individuals with ASD are usually accompanied by other conditions, including epilepsy, intellectual disability (ID), sleeping disorders, anxiety, motor-control difficulties, attention deficit hyperactivity disorders, and gastrointestinal disorders (3, 4).

Currently, the exact etiologies of ASD are still largely unknown, and no hypothesis can completely explain the etiology and pathology of ASD. However, it is generally accepted that ASD is caused by genetic and environmental factors or their interaction (5). An increasing number of studies from patients and animal models of ASD suggested that the abnormal synaptic structure and function changes are closely related to the onset of ASD (6–8). Therefore, ASD is often conceptualized as a synaptic disorder or called “synaptopathy” (9, 10), indicating that the etiology of ASD, at least in part, is attributed to synaptic abnormalities, and targeting ameliorating the dysregulation of synaptic structure and function may be a promising method to promote ASD clinical rehabilitation.

However, despite the high prevalence of ASD, no effective pharmacological treatments to cure it have been proposed (11). Clinically, the drugs are used mainly to improve co-occurring behaviors or problems but not ASD (1). Over the decades, numerous evidence has been demonstrated that different exercise interventions positively influence physical functions for people with ASD, such as improved motor skill performance (12) and reduced body mass index (13). Apart from the physical benefits, exercise intervention has been shown to improve social skills (14) and cognitive functions (15), as well as reduce maladaptive behaviors (16) and stereotypic behaviors (17). Meanwhile, improvements in academic engagement (18), sleep quality (19), and emotional problems (20) among children with ASD have also been reported. These findings suggested that exercise intervention, as a non-pharmacological treatment, is effective for ASD symptoms, particularly if these interventions are introduced early in life. However, research investigating the underlying possible molecular and cellular biological mechanisms is still insufficient, and further research is necessary.

Hence, in this review, we summarized the characteristics of synaptic structural alternations in the context of ASD and discussed their possible roles in the pathogenesis of ASD. Then, we explored the molecular and cellular mechanisms behind these abnormal changes from the perspective of ASD-associated genetic mutation, signal pathway, and environmental factors. In addition, we reviewed the effects of an exercise intervention on individuals with ASD. We listed the possible neurobiological mechanisms behind it to gain a better understanding of the synaptic impairments underlying ASD and the mechanisms of improvement using exercise intervention.

## 2. Characteristics of synaptic structural alterations in ASD brains

Much remains to be understood about the characteristics of synaptic structural alterations in ASD brains. The pathologies of several neuropsychiatric disorders, such as ASD, ID, Alzheimer's disease (AD), and schizophrenia, present with alterations in synapses and the dendritic spine (spine) (8). In ASD, the first report concerning this topic was proposed by Williams et al. (21). They found an apparent reduction in the spine density on the dendrites of some pyramidal neurons in adolescents and one of the adult patients. However, the morphology was normal (21). Subsequently, other studies of human brains indicated that the hippocampus and prefrontal cortex presented with decreased dendritic branching and spine density (22, 23). However, different results exist, an increased mean spine density was found in the cortical pyramidal neurons of ASD subjects compared to those age-matched subjects (24). Tang et al. (25) found that layer V pyramidal neurons in the temporal lobe of ASD individuals showed a higher dendritic spine density than the brain samples in control subjects. However, reduced spine elimination from the age of children to adolescence indicates synaptic pruning deficits. Notably, a study reported that the high density of spines was most commonly found in ASD individuals with more severe cognitive deficits (8). Thus, future studies of human neuropathologies should strive to understand the degree of correlation between the severity of cognitive deficits and spine dysmorphogenesis. The variations in findings across studies may be attributed to some confounding factors, such as the postmortem interval, symptomatic heterogeneity, and patient medical history (8). Together, these findings from human brains revealed that the abnormal changes in spine density are closely related to the pathologies of ASD. Thus, it is possible that aberrant alterations in the spine, the increased or decreased density, contribute to the disruptions of specific neural circuits, which, in turn, may underlie the socio-cognitive impairments of this disorder. The brain samples are typically collected from deceased adults, an endpoint of the disease process. Therefore, when abnormal synapse and spine changes occurring in ASD remain unknown, thereby emphasizing the need to develop better diagnostics and provide timely interventions.

To explore the specific disease-causing factors on neuropathology, synaptic function, and behavioral outcomes more thoroughly, ASD animal models provide valuable tools. Further evidence of spine pathology from ASD animal models underscores the importance of these structural perturbations in the pathogenesis of ASD and comorbid disorders. The detailed data have been described by Martínez-Cerdeño (26) and Forrest et al. (27). Overall, structural alterations of the spine are a common feature of ASD. Specifically, too much or too little spine density in ASD affects synapse and circuit-level connectivity functional changes.

## 3. Molecular mechanisms of the structural alterations of the synapse/spine in ASD

**Figure 1:**
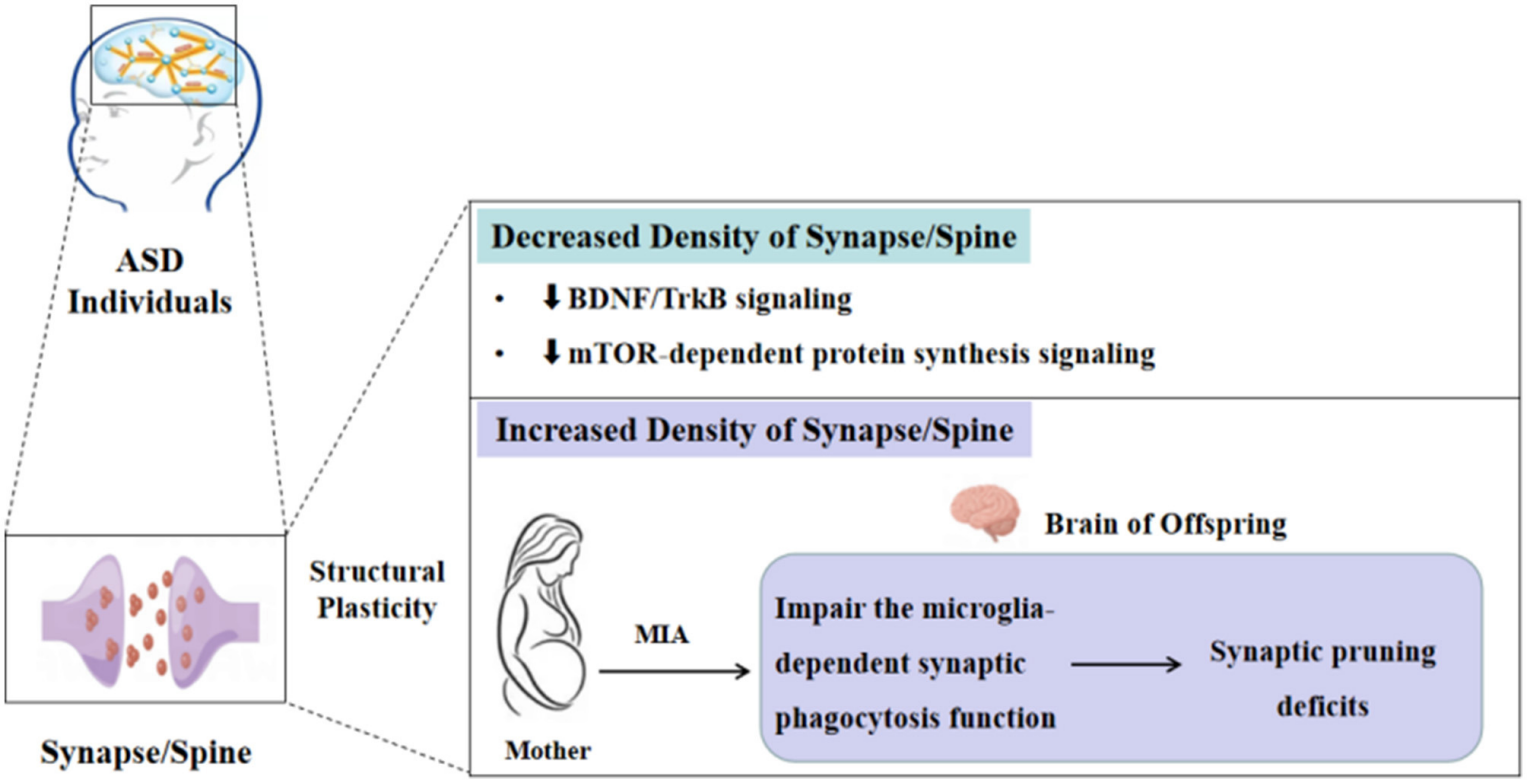
Molecular mechanisms of the synapse/spine density alterations in ASD.

The phenotypes of decreased and increased density of the spine in ASD individuals and different animal models have been widely described, but the underlying molecular mechanisms of these changes in spine density in ASD remain unclear and need to be explored further. Given the complexity and heterogeneity of ASD etiology, currently, a united molecular biology theory illustrates that all ASD cases have been lacking. In the present review, we will discuss the possible molecular mechanisms in certain ASD types on spine density changes from the perspective of brain-derived neurotrophic factor/tropomyosin receptor kinase B (BDNF/TrkB), mechanistic target of the rapamycin (mTOR)-dependent protein synthesis signaling pathway, and microglia-dependent synaptic pruning (Figure 1).

### 3.1. Decreased density of the synapse/spine in ASD

#### 3.1.1. Downregulation of the BDNF/TrkB signaling pathway

It is well known that BDNF, a member of neurotrophic factors, plays an important role in synaptic development and plasticity, which is widely expressed in the hippocampus and cortex of the central nervous system (CNS) (28). The BDNF in the brain can be released into the blood through the blood–brain barrier. It is positively correlated with the serum BDNF levels, that is, the BDNF levels in peripheral serum can reflect the changes in the BDNF levels in the brain. The downstream signaling pathways, including mitogen-activated protein kinase/extracellular signal-regulated kinase (MAPK/ERK), phosphoinositide-3 kinase (PI3K), and phospholipase C (PLC)-gamma, are activated after BDNF combines with the TrkB, a high-affinity receptor for BDNF (29), thereby regulating the progress of growth and survival of neurons, spine formation, and synaptic plasticity (30). Furthermore, a series of studies have shown that BDNF is crucial for the density and morphology of the spine, and these effects of BDNF on spine density and morphology occur in a TrkB-dependent manner (31). Inhibiting BDNF/TrkB signaling will reduce spine number and change spine morphology toward a more immature phenotype (32). Therefore, applying BDNF as a therapeutic agent to treat the associated symptoms in many disease models has shown beneficial effects *in vitro* and *in vivo* (31).

Downregulation of the BDNF/TrkB signaling pathway has been reported to be closely associated with ASD. BDNF is one of the neuroprotective substances, a reduction in BDNF release is observed under pathological conditions. Therefore, BDNF has been considered a biomarker for different brain pathologies (31). A study assessing the BDNF levels in serum found that the BDNF levels are lower in children with ASD than in normal controls aged 30–42 months (33). Another study discovered significantly decreased protein levels of full-length TrkB in ASD versus control subjects (34). However, only a few studies in animal models have investigated the association between BDNF levels and ASD, and immediate evidence from tissue pathology alterations of the spine with the change of BDNF expression is lacking. Consistent with the result for ASD individuals, Sprague–Dawley offspring that received valproic acid (VPA) during pregnancy displayed a notably reduced BDNF expression in the dorsomedial prefrontal cortex and hippocampus (35). Several reports revealed decreased spine density and changes in spine morphology with a reduction of BDNF levels in methyl-CpG binding protein 2 (*MECP2*) KO mice (36). Notably, overexpression of BDNF rescues several cellular and behavioral deficits. These effects of BDNF on the spine may depend on the TrkB receptor, thus stimulating the downstream signaling pathways (36, 37). Recently, applying small molecules or small compounds to induce BDNF expression or activate the TrkB receptor has been demonstrated to be a beneficial strategy for neuroprotection and neuroregeneration (29). These results indicate that impaired BDNF/TrkB signaling is involved in the pathogenesis of ASD, and targeting for inducing BDNF expression or activating the TrkB receptor may be a promising strategy to rescue ASD symptoms.

#### 3.1.2. Downregulation of the mTOR-dependent protein synthesis signaling pathway

Aberrant mTOR-dependent synaptic protein synthesis may represent one possible pathway leading to ASD-like behaviors (38). mTOR, as a signaling “hub” that integrates neuronal activity and a variety of synaptic inputs, is involved in multiple cell biological processes, including protein synthesis, transcription, actin dynamics, neuronal morphology, and autophagy. mTOR contains two structurally and functionally distinct complexes, namely mTOR complex 1 (mTORC1) and mTOR complex 2 (mTORC2) (39). Regulation of synaptic protein synthesis by mTOR depends mainly on the phosphorylation of mTORC1 main downstream effectors eukaryotic initiation factor 4E-binding proteins (4E-BPs) and p70S6 kinases (S6K1 and S6K2) (39), suggesting that mTORC1 activation is necessary for synaptic protein synthesis. In addition, the mTOR signaling pathway also plays a great role in synaptic plasticity in the CNS (40). Therefore, it is not surprising that dysregulated mTOR signaling pathway is implicated in many neurodevelopmental and neuropsychiatric disorders, such as epilepsy, ASD, ID, and AD.

Reduced mTORC1 activity has been found in certain ASD models. In *MECP2* mutation-causing ASD models, it was found that both protein synthesis and mTORC1 activity were reduced, this may be connected with decreased levels of BDNF leading to the reduction in the activity of the PI3K/mTORC1 pathways (40). Evidence from phosphoproteomic studies of neurons in the rodent models of the Phelan-McDermid syndrome with the SH3 and multiple ankyrin repeat domains protein 3 (*SHANK3*) knockdown revealed reduced phosphorylation levels of Akt and mTORC1 (41). Most of the knowledge on the changes in the mTOR signaling pathway emerged mainly from studies in syndromic ASD and a few studies in idiopathic ASD. Nicolini et al. (34) revealed a reduction of phosphorylated and total Akt, mTOR, 4E-BP1, and phosphorylated S6 protein in VPA rats. It is important to mention that the downregulation of the mTOR signaling pathway is accompanied by decreased spine density and typical autistic behaviors in these ASD models. These results indicate that dysregulated mTOR-dependent protein synthesis signaling pathway is one of the reasons for decreased spine density.

### 3.2. Increased density of the synapse/spine in ASD

During normal development, synaptic and spine density increases rapidly from birth to early development, reaching its peak during adolescence, then subsequently decreasing from adolescence to adulthood. This progress of decline is called synaptic pruning or synapse elimination (27, 42), which is crucial for the refinement of neuronal circuits and normal brain function by removing some unnecessary synapses and maturing the morphology and function of the remaining synapses (42). However, multiple studies have reported that increased spine density has been found in certain ASD individuals and mouse models, which may be attributed to insufficient synaptic pruning (25, 42), but the molecular mechanisms underlying synaptic pruning disorder in ASD have not been fully elucidated.

Recently, the role of microglia in mediating the synaptic pruning deficits of ASD has been gradually revealed. Microglia, as brain-resident immune cells, not only survey the brain microenvironment with highly motile processes but are also implicated in the formation of neural circuits during development *via* synapse engulfment; therefore, the disruption of the microglia-dependent synapse elimination is thought to be closely connected with some neurodevelopmental disorders including ASD (42). In addition, maternal immune activation (MIA) has been proven to disrupt microglial properties (such as number, morphology, and cytokine expression) and impair the microglia-dependent synaptic phagocytosis function (43, 44). In the MIA mouse model, activation of microglia and reduced phagocytic function of microglia in the adult offspring accompanied by social abnormality are observed (42). Similar to another report, the results have shown that MIA mice offspring at postnatal day 60 exhibited an increased density of hippocampal CA3 synapses. The authors further determined that synaptic surplus in the MIA mice offspring may be induced by deficits in synapse engulfment by microglia (45). Notably, these changes can be improved after treatment with minocycline. The aforementioned results suggested that MIA can affect the phagocytic function of microglia, leading to increased density of synapse and spine in ASD, thereby resulting in neuronal network deficits.

## 4. Beneficial effects of exercise intervention on ASD symptoms

It is widely accepted that treating patients with ASD should be aimed at improving the quality of life and reducing family suffering (46). At present, although there are no known medications that can effectively treat the core symptoms of ASD (1), especially social and communication deficits, some interventions can help ASD individuals to promote their quality of daily life (46). As a non-pharmacological intervention, exercise intervention has many beneficial roles in improving related symptoms in individuals with ASD. It has also been employed as an adjuvant therapy measure to treat ASD children within usual care treatments, and exercise intervention can be considered an evidence-based practice for school-aged children with ASD (47). Specifically, Yang et al. (14) found that a 12-week mini-basketball training program improved the social communication and the executive control network in preschool children with ASD. These effects may be related to the enhanced functional connectivity between the right cerebellum and the left inferior frontal gyrus in the experimental group. Remarkably, among various exercise intervention programs (e.g., swimming, jogging, yoga, and basketball), the team sports such as basketball intervention are considered a promising strategy for improving social deficits compared to individual sport (e.g., jogging), because it requires individuals to make decisions and communicate with others while playing such team sports (48–50). Regarding stereotypical behaviors, aerobic exercise has been shown to reduce this phenotype (51). One study found that low- to moderate-intensity exercise produces significant and large reductions in these behaviors (17). These studies establish that exercise intervention can positively impact the core symptoms of ASD.

**Figure 2:**
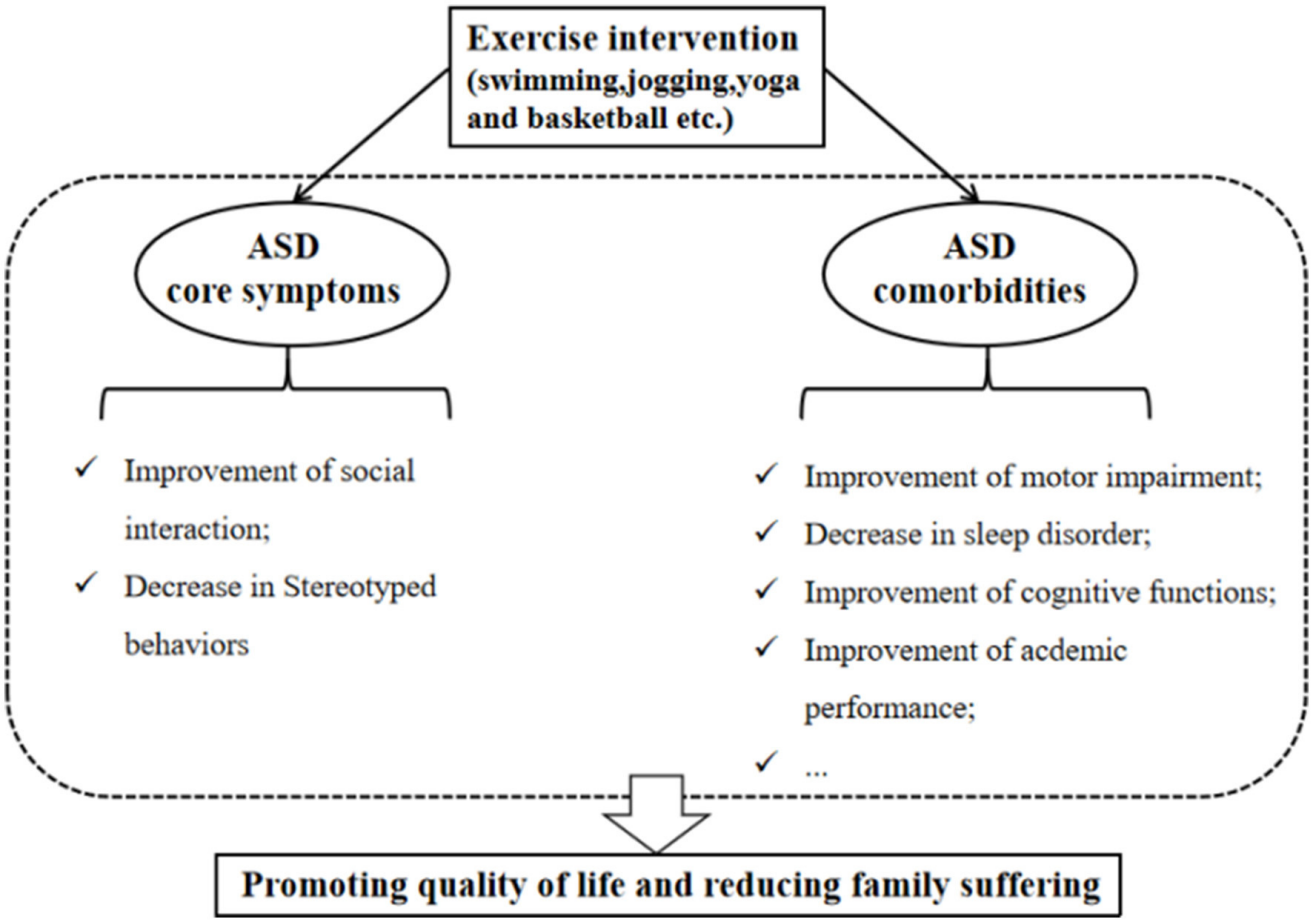
Beneficial effects of an exercise intervention on ASD symptoms.

Exercise intervention can not only positively impact the primary symptoms of ASD but also contribute to the improvement of ASD comorbidities (Figure 2). Research suggested that reduced participation in physical activity and a higher incidence of obesity among ASD children than peers may be related to their symptoms (52, 53). Conversely, reduced participation may limit the opportunities to communicate with peers, leading to further developing obesity and ASD symptoms (11). Therefore, it is essential to encourage ASD children to engage in physical activity. Moreover, sleep difficulties in children with ASD are widespread (54). Compared to ASD children with lower physical activity levels, those ASD children with higher physical activity levels display fewer sleep problems and better overall sleep quality, suggesting that engaging in physical activity contributes to improving sleep disorders (55). Motor skill impairment is a limitation to participation in physical activity, Rafie et al. (56) found that different games (e.g., ball games, fun games, and orienteering games) all improve the perceptual motor skills of ASD adolescents. Interestingly, exercise intervention (e.g., jogging) can also enhance the academic performance of ASD children, which reflects the improvement of attention (11).

Similarly, the positive effects are also observed in ASD animal models. In the VPA-induced animal model of ASD, several reports indicated that treadmill exercise is associated with improving aggressive behavior, spatial learning memory, and motor dysfunction (57–59). In another ASD animal model, early swimming intervention can lessen the core symptoms of ASD rats caused by *SHANK3* knockout (60). Andoh et al. (45) found that voluntary wheel-running exercise reverses the ASD-like behaviors in offspring after MIA.

Altogether, appropriate exercise intervention can improve ASD-associated symptoms in ASD individuals and animal models and enhance the social adaptability of ASD individuals. However, given the complexity of the etiology and heterogenicity of phenotype in ASD, an acceptable standardized exercise intervention program has not yet been formulated to control all symptoms. Consequently, personalized exercise intervention programs should be designed depending on the various symptoms and severity of ASD individuals in future.

## 5. Possible molecular mechanisms of exercise intervention in the improvement of ASD symptoms

Many experimental studies have widely affirmed the beneficial effects of an exercise intervention on ASD, but few have explored the response of the underlying molecular mechanisms to exercise intervention. In recent years, the disorder of ASD has been recognized as “synaptopathy,” characterized by abnormal synaptic structure and functional changes. Specifically, an increased or reduced density of synapses and spine can be observed in the brain of ASD individuals and animal models. In this section, we discuss the underlying possible molecular mechanisms of exercise intervention that improve ASD symptoms by regulating the structural plasticity of synapses and the spine.

### 5.1. Exercise intervention can regulate the structural plasticity of the synapse/spine

Exercise intervention can induce alterations of synaptic function by regulating the structural plasticity of synapses and the spine, ultimately leading to improved behaviors. However, due to limited technical means and other reasons, it is currently impossible to collect evidence from human synaptic changes in a non-invasive manner. Therefore, these results are mainly obtained from animal research. A recent study is the first time that the promotion of exercise intervention on synaptic function has been confirmed in human studies, the researchers analyzed brain tissues from 404 decedents and found that greater physical activity during late life is related to higher presynaptic protein levels, indicating that their synaptic integrity is better (61). Chen et al. (62) identified that chronic treadmill exercise could enhance synaptogenesis and synaptic transmission and increase neuron activity and axonal myelination in mice models, leading to improved motor learning. In addition, physical exercise also promoted spinogenesis in the mouse barrel cortex (63). Exposure to cocaine can impair cortical plasticity and motor learning. At the same time, a study reported that 1-week treadmill exercise training could rescue motor-learning impairments in mice by improving spine formation, synaptic transmission, and spontaneous activities of cortical pyramidal neurons (64). Moreover, exercise intervention has been shown to prevent the spine loss of striatal medium spiny neurons in the mouse model of Parkinson's disease (65).

It is worth noting that exercise intervention can not only improve learning and cognition by promoting synaptogenesis but also decrease the density of synapses by regulating microglia-dependent synaptic pruning, leading to the optimization of the neural circuits in the brain. In 2019, a study published in *Cell Reports* reported that voluntary wheel-running exercise decreased the density of hippocampal CA3 synapses in the MIA-affected offspring by promoting the synaptic pruning by microglia and reversed the ASD-like behaviors (45). This study is also considered a pioneering study because it successfully manipulated microglial function in ASD through a non-invasive method (42). In another report, evidence from Golgi-stain suggested an increased hippocampal spine density and impaired short-term memory performance in sleep-deprived mice. However, in exercise groups, voluntary exercise normalized the density of the spine induced by sleep deprivation and improved short-term memory. These effects may be caused by improving microglial phagocytic function in sleep-deprived mice (66).

### 5.2. Exercise intervention promotes synaptic formation by regulating the BDNF/TrkB signaling pathway

Downregulation of the BDNF/TrkB signaling pathway with the decreased density of synapse and spine has been observed in certain ASD animal models, such as Rett syndrome (RTT) and VPA models. Therefore, upregulating the BDNF/TrkB signaling pathway may be a feasible treatment for ASD. In the RTT mouse model, environmental enrichment has been shown to improve motor coordination, learning deficits, and anxiety in *Mecp2*^+/−^mice, which may depend on increased BDNF expression (67). Similar to the effect produced by environmental enrichment, 7 days of voluntary wheel-running exercise can promote hippocampal BNDF expression in rats (68). Results from *in vivo* two-photon imaging indicated that stress could induce spine loss in the barrel cortex and working memory impairment in mice. Physical exercise suppressed the spine elimination in the barrel cortex and improved working memory by promoting the BDNF/TrkB expression. To certify the role of the BDNF/TrkB signaling pathway in physical exercise-regulated spine plasticity and working memory, researchers further applied ANA-12 to block the TrkB pathway. They found that these beneficial effects of physical exercise were abolished (63). These results suggested that exercise intervention can improve the structural plasticity of synapses and the spine by regulating the BDNF/TrkB signaling pathway.

### 5.3. Exercise intervention promotes synaptic formation by regulating the mTOR-dependent protein synthesis signaling pathway

Physical exercise can improve behavioral performances (e.g., learning and memory). Previous studies mainly converged on the expression of BDNF and insulin-like growth factor-1 (62, 69). Chen et al. (62) demonstrated for the first time that exercise training could improve motor skill learning in mice by activating the mTOR-S6 pathway, which is crucial for spinogenesis and synaptic transmission. Previous reviews show that a reduced expression of the mTOR-dependent protein synthesis signaling pathway is observed in certain ASD models (39, 40). Therefore, we proposed that the improvement of ASD symptoms induced by exercise intervention may be caused by the increased density of synapses and the spine by regulating the mTOR-dependent protein synthesis signaling pathway.

### 5.4. Exercise intervention promotes synaptic elimination by improving synaptic pruning deficits

Deficits of synaptic pruning caused by microglial dysfunction may underlie the increased density of synapses in ASD, suggesting that microglia may be a therapeutic target for ASD (70). This section focuses on synaptic elimination by improving synaptic pruning deficits in ASD brains. Specifically, we discuss the possibility that exercise intervention alleviates ASD behaviors by regulating microglial phenotypes and inducing synaptic competition by microglia.

#### 5.4.1. Exercise intervention regulates microglial phenotypes by controlling pro-inflammatory and anti-inflammatory cytokines

Maternal immune activation has been shown to activate the microglia releasing inflammatory cytokines and impairing the synaptic phagocytic function of microglia, suggesting the alterations of microglial phenotypes and that these changes are accompanied by the occurrence of ASD-like behaviors (71). Hence, we proposed how microglial phenotypes are regulated may be a feasible pathway to improve ASD symptoms.

On the one hand, exercise intervention can inhibit microglial activation by promoting the expression of anti-inflammatory cytokines. Several studies have shown that some anti-inflammatory cytokines, such as the cluster of differentiation-200/CD200 receptor and interleukin-10 (IL-10), and triggering receptor expressed on myeloid cell-2 and vitamin D3 can inhibit microglial activation and that exercise intervention can promote the expression of these proteins (72). For example, compared to the control group, treadmill training over 10 consecutive days increased the cytokines levels of IL-10 in the hippocampal formation of aged rats (73). Moreover, when IL-10 interacts with its receptor on microglia, it can enhance the expression of suppressor of cytokine signaling, resulting in the inhibition of microglial activation (74). On the other hand, exercise intervention can also inhibit microglial activation by decreasing the expression of pro-inflammatory cytokines. Interleukin-1β (IL-1β), interleukin-18 (IL-18), and tumor necrosis factor-α (TNF-α) are considered the major pro-inflammatory cytokines in the CNS, and these cytokines can promote inflammatory response by increasing the expression of the inflammasome (75). In the APP/PS1 mice model of AD, 12 weeks of treadmill exercise can inhibit microglia-mediated neuroinflammation and oxidative stress by reducing the expression of IL-1β and TNF-α (76). Exercise intervention also decreased levels of IL-1β and IL-18 in the hippocampus of ovariectomized rats and suppressed the NLRP3 inflammasome and microglial activation (77). Accordingly, it is reasonable to infer that exercise intervention suppressing the activation of microglia and sustaining its synaptic phagocytic function may depend on increased anti-inflammatory factors and reduced pro-inflammatory factors.

#### 5.4.2. Exercise intervention induces synaptic competition by selectively enhancing partial synaptic activity

It has been confirmed that exercise intervention can ameliorate insufficient synaptic pruning and aberrant behaviors in ASD mice by preferentially engulfing the weaker synapses by microglia, indicating that microglia can decipher the strength of synaptic activity (45). However, the underlying molecular mechanisms of exercise intervention enhancing the elimination of the weaker synapses by microglia in ASD are unclear and may depend on exercise-induced synaptic competition. On the one hand, newly formed and growing spines can drive the shrinkage and elimination of relatively weak and inactive neighboring spines (78). Oh et al. (79) found that the structural potentiation of the individual spine could be stimulated by high-frequency glutamate, which drives the shrinkage of nearby inactive spines, suggesting that the competition between spines is activity-dependent. On the other hand, the lower active synapses can be tagged by complement molecule C1q and its downstream component C3, which can be further recognized by the C3 receptor, CR3. Finally, microglia phagocytose these synapses (42).

The competition between synapses in response to exercise intervention may be modulated by the expression of neurotrophic factors, such as BDNF, which is enriched in the mossy fiber boutons of the hippocampus (80) and can increase the maturation and stabilization of synapses and the spine (31). Furthermore, the release of BDNF is activity dependent (81), and exercise can upregulate the expression of BDNF by enhancing neuronal activity (32). Thus, it is possible that exercise intervention can promote the release of BDNF from mossy fiber boutons by activating the hippocampal dentate granule cells, resulting in a portion of synapses being strengthened and, subsequently, synaptic competition.

**Figure 3:**
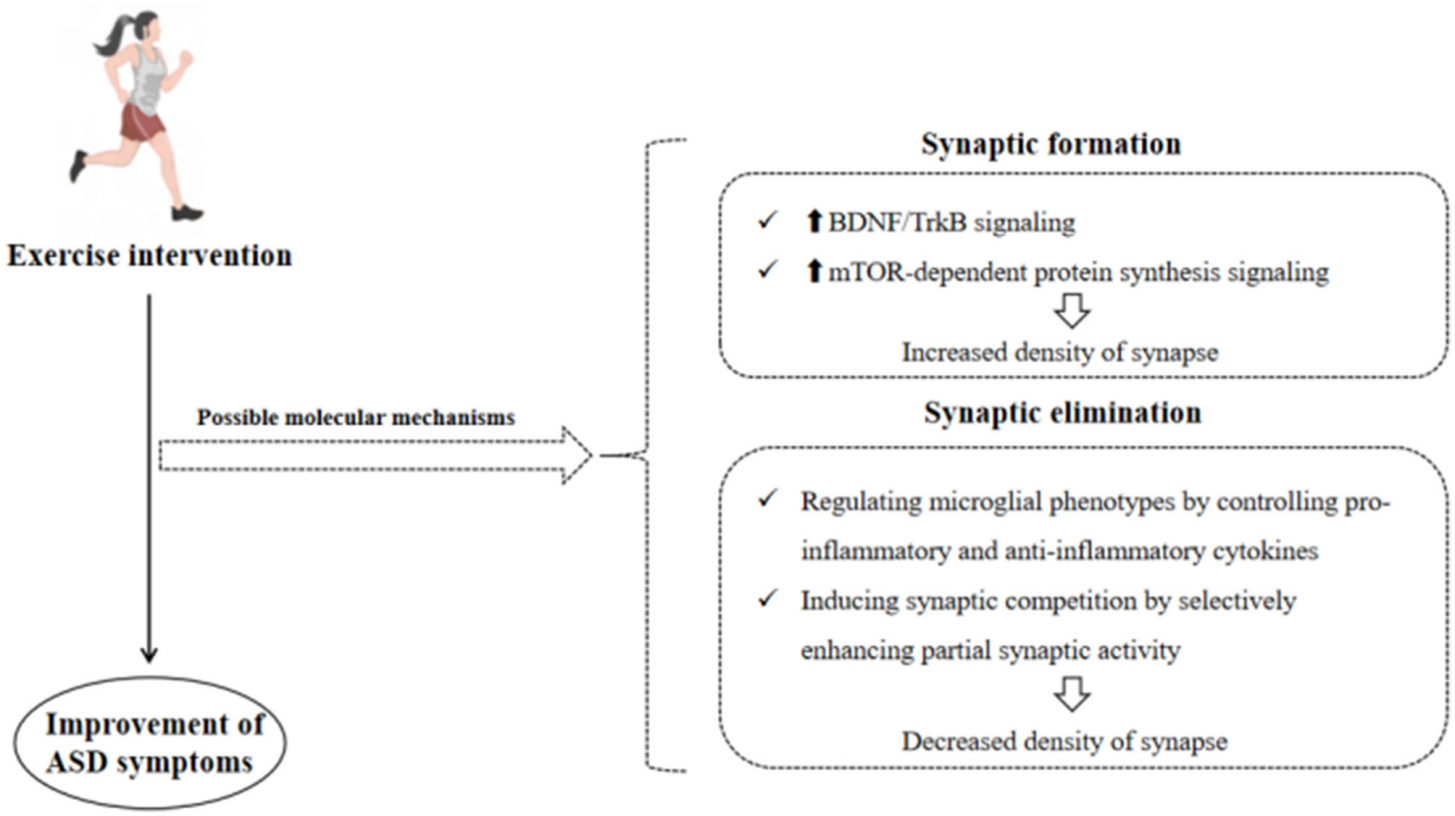
Possible molecular mechanisms of exercise intervention in improving ASD symptoms.

In summary, exercise intervention might regulate the structural plasticity by controlling the BDNF/TrkB and mTOR signaling pathway as well as microglial function, thereby improving ASD symptoms (Figure 3).

## 6. Conclusion and outlook

In this review, we summarized the structural alterations of the synapse/spine in ASD and its molecular mechanisms. Importantly, we also discussed that exercise intervention, as a cost-effective therapeutic strategy, significantly improves ASD symptoms. Finally, we mainly emphasized the possible underlying molecular mechanisms from the perspective of microglia-dependent synaptic pruning. Admittedly, these results concerning molecular mechanisms are from animal experiments. Although animal models are a powerful tool for exploring synaptic or cellular physiology, ultrastructure, and biochemistry alterations, species-specific differences might limit direct predictions in humans. Thus, results from clinical trials are required to provide reliable information on the possibility of alleviating the disorder.

Synaptic deficits underlie the pathogenesis of the disorder of ASD. Exercise intervention has been shown to play a significant role in improving ASD symptoms. However, few studies have explored the underlying synapse-related molecular mechanisms. Currently, the existing report is only from the perspective of animal experiments. The report has demonstrated that voluntary wheel-running exercise could ameliorate core symptoms of ASD mice induced by MIA. These effects may be caused by regulating microglia-dependent synaptic pruning deficits, but many questions remain. For example, the molecular mechanisms of exercise intervention improving the developmental synaptic pruning disorder are unclear. The present studies mainly converge on microglia-mediated synaptic pruning. Recently, astrocyte has also been shown to be involved in synaptic pruning. However, the molecular mechanisms for whether or how it mediates the process of exercise intervention regulating synaptic pruning have not been reported. In microglia-dependent synaptic pruning, the classical complement system is an important pathway to mediate microglia to recognize low active synapses, leading to synaptic elimination. However, how the classical complement system works during exercise intervention regulating synaptic pruning remains to be demonstrated. Further clarification of these questions may lead to the elucidation of the molecular mechanisms for improving ASD symptoms through exercise intervention.

## Author contributions

WZ drafted the work. XL collected the information. GD, LZ, and KL substantively guided and revised it. All authors contributed to the conception, design of the work, collected the information, analyzed data used in the systematic review, read, approved the submitted version, agreed to be personally accountable for the authors' contributions, and to ensure the accuracy and integrity of the work.
